# The gliadin-CFTR connection: new perspectives for the treatment of celiac disease

**DOI:** 10.1186/s13052-019-0627-9

**Published:** 2019-03-21

**Authors:** Luigi Maiuri, Valeria R. Villella, Valeria Raia, Guido Kroemer

**Affiliations:** 1 Department of Health Sciences, University of Eastern Piedmont, Novara, Italy; 2 European Institute for Research in Cystic Fibrosis, San Raffaele Scientific Institute, Milan, Italy; 3 Regional Cystic Fibrosis Center, Pediatric Unit, Department of Translational Medical Sciences, Federico II University Naples, Naples, Italy; 4 Equipe11 labellisée Ligue Nationale contrele Cancer, Centre de Recherche des Cordeliers, Paris, France; 5 INSERM U1138, Centre de Recherche des Cordeliers, Paris, France; 6 Université Paris Descartes, Paris, France; 7 Metabolomics and Cell Biology Platforms, Institut Gustave Roussy, Villejuif, France; 8 Pôle de Biologie, Hôpital Européen Georges Pompidou, AP-HP, Paris, France; 9 Karolinska Institute, Department of Women’s and Children’s Health, Karolinska University Hospital, 17176 Stockholm, Sweden

**Keywords:** CFTR, Celiac disease, Gliadin

## Abstract

Familial loss-of-function mutations of the gene coding for the cystic fibrosis transmembrane conductance regulator (CFTR) channel protein cause cystic fibrosis (CF), the most frequent inherited life-threatening disease in the Caucasian population. A recent study indicates that the gluten/gliadin-derived peptide (P31–43) can cause CFTR inhibition in intestinal epithelial cells, thus causing a local stress response that contributes to the immunopathology of celiac disease (CD). Accordingly, an increased prevalence of CD has been observed in several cohorts of CF patients. CD is characterized by a permanent intolerance to gluten/gliadin proteins occurring in a proportion of susceptible individuals who bear the human leukocyte antigen (HLA) DQ2/DQ8. In CD, perturbations of the intestinal environment, together with the activation of the innate immune system by P31–43, are essential for rendering other immunodominant gliadin peptide fully antigenic, thus triggering an adaptive immune response with an autoimmune component. P31–43-induced CFTR inhibition elicits the danger signals that ignite the epithelial stress response and perturb epithelial proteostasis. Importantly, potentiators of CFTR channel gating, such as the FDA-approved drug Ivacaftor, prevent P31–43 driven CFTR inhibition and suppress the gliadin-induced stress response in cells from celiac patients, as well as the immunopathology developing in gliadin-sensitive mice. Thus, CFTR potentiators may represent a novel therapeutic option for celiac patients.

## Background

The intestinal mucosa constitutes the first-line defense against dietary or microbial challenges and usually avoids unwarranted inflammatory reactions in response to non-self-antigens by promoting oral tolerance [1]. The capacity of the mucosal immune system to neutralize harmful luminal challenges, can be subverted by exogenous triggers, such as viruses [2], or conditions in which still undefined inherited or acquired cell-autonomous factors favor an intestinal pro-inflammatory state. Cystic fibrosis (CF), the most frequent monogenic lethal disease in the Caucasian population [3], is the quintessential example of a disease in which cell-autonomous triggers favor antigen mishandling by the intestinal mucosa. Indeed, in the CF intestine, two unfavorable events determine an inadequate cellular and humoral immune response to food components, (i) the increased antigenic load due to pancreatic insufficiency and (ii) the constitutive chronic intestinal inflammation due to loss-of function-mutations in the CF transmembrane conductance regulator (CFTR) gene [4, 5]. Accordingly, CF patients often manifest increased levels of antibodies against alimentary antigens, including anti-gliadin IgA antibodies, increased intestinal permeability, elevated levels of fecal calprotectin, shifts in the intestinal microbiota, and increased intestinal permeability [5–7]. Importantly, CF patients manifest a threefold increase in the prevalence of celiac disease (CD) [8, 9] a permanent intolerance to gluten/gliadin proteins that occurs in a proportion of susceptible individuals who bear the human leukocyte antigen (HLA) DQ2/DQ8 [10–12]. Of note, a prevalence as high as ~ 4% of positive anti-TG2-IgA autoantibodies, a serological marker of CD, has been reported in several cohorts of CF patients [5–7], even in the absence of villous damage, the hallmark of CD. Thus, there is an epidemiological link between CF and CD.

## CF and CF, a mysterious connection

The unexpected link between CF and CD, the gluten enteropathy triggered the hypothesis that CFTR might be involved in gluten sensitivity. Mounting evidence supports this hypothesis. Indeed, CFTR is not only an anion channel relevant to CF, but is also a hub protein that orchestrates the proteostasis network of epithelial cells, including enterocytes, thus regulating adaptation to cell-autonomous or external stress [13–19]. Loss of CFTR function causes increased generation of reactive oxygen species (ROS) and persistent activation of tissue transglutaminase (TGM2) [13–19], which targets several TGM2 substrates, including the autophagy-relevant Beclin 1 protein (BECN1), hence suppressing autophagy. Moreover, BECN1 targeting by TGM2 results in the functional sequestration of BECN&-asssociated phosphatidylinositol 3-kinase catalytic subunit type 3 (PIK3C3) with reduced availability of the PIK3C3 product phosphatidyl-inositol-3-phosphate *(PtdIns3P)* at early endosomes, thus impairing endosomal maturation and trafficking [13, 14].

In addition, TGM2 activation leads to increased nuclear translocation of nuclear factor kappa-light-chain-enhancer of activated B cells (NF-κB) owing to TGM2 targeting of the NF-κB inhibitor alpha (NFKBIA) [13, 15, 18]. NF-κB activation then leads to increased levels of pro-inflammatory cytokines, including interleukin (IL)-17A, IL-21 and IL-15, a master cytokine involved in gut homeostasis [20–22] as well as IL-1β (downstream of both NFκB and the NLRP3 inflammasome).

Interestingly, these consequences of CFTR malfunction are reminiscent of those induced by some gliadin fractions in celiac intestine [12, 23, 24]. Indeed, after gluten ingestion, two major peptides, the 25-mer and the 33-mer, remain undigested and induce innate immunity activation and adaptive Th1-mediated immune responses, respectively. Some peptide fractions, such as P31–43, a fragment of to 25-mer, are capable of triggering an enterocyte stress response that is accompanied by TGM2 activation, derangement of endosomal trafficking, increased NF-κB nuclear translocation and consequent IL-15 upregulation [10, 12, 23–26].

In the intestine from CD patients, the perturbation of the local environment with activation of the innate immune response by the 25-mer (or its fragment P31–43), is required to allow the immune-dominant gliadin peptides (33-mer or its fragment P57–68) to elicit a Th1 and antibody responses [10, 12, 23–26]. However, how P31–43 can induce an epithelial stress response has remained elusive, although putative additional factors as reovirus [2] or yet-to-be defined cell-autonomous events are required to provide the danger signal that can ignite P31–43 induced epithelial stress [10, 12, 25].

## CFTR, an unforeseen surface receptor for gliadin

Given the consequences of CFTR malfunction observed in CF, the intriguing question arises as to whether CFTR inhibition represents the cell-autonomous event that mediates gliadin-induced stress response in CD.

In a recent study [27] we demonstrated a molecular and functional interaction between P31–43 and CFTR. By using a multifaceted experimental approach including computer-assisted calculations, plasmon surface resonance, co-immunoprecipitation, fluorescent confocal microscopy and electrophysiological measurements of CFTR dependent chloride currents, we demonstrated that P31–43 binds to, and reduces the ATPase activity of, the nuclear binding domain 1 (NBD1) of CFTR, thus impairing CFTR channel function. The CFTR inhibitory effect of gliadin was confirmed in three different mouse models of gliadin-sensitivity [27].

CFTR oscillates between the open and closed conformational states of the chloride channel [4, 28]. Notably, P31–43 can only efficiently bind to NBD1 when it is in the closed state, leading to the block of its gating function [27]. Moreover, increasing the probability of CFTR channel opening by means of pharmacological “potentiators” of CFTR channel gating prevent CFTR inhibition by P31–43, as well as the P31–45 induced enterocyte stress response [27]. This could have clinical relevance as a CFTR potentiator, VX-770 (Ivacaftor, Kalydeko) is already FDA-approved for the treatment of CF patients bearing CFTR mutations with gating defects.

## CFTR, a sensor of stress

As mentioned before, CFTR inhibition drives the activation of TGM2, a pivotal enzyme in CD pathogenesis as it deamidates P57–68, thus dramatically increasing its antigenicity [12, 23, 24, 29]. Moreover, activated TGM2 engages in multiple vicious cycles that aggravate the disease, in thus far that it crosslinks the complex formed by CFTR and P31–43, thus creating a trimolecular complex (CFTR, P31–43, TGM2) that renders CFTR inhibition irreversible. Moreover, TGM2 inactivates BECN1, thereby inhibiting autophagy, interfering with endosomal maturation and trafficking and driving inflammasome activation and IL-15 production [27].

Of note, Ivacaftor, as well as another CFTR potentiator, Vrx-532, are highly effective in preventing all these P31–43/gliadin driven manifestations. Importantly, the protective effect of Ivacaftor on intestinal epithelial cells, was abrogated when CFTR was genetically depleted, thus confirming that Ivacaftor protects enterocytes from the detrimental effects of gliadin through potentiating CFTR channel function [27].

## CFTR inhibition orchestrates the pathogenic response to gliadin

In vivo experiments, in which 3 different mouse models of gliadin sensitivity (including non-obese diabetic (NOD) mice) were treated with Ivacaftor and orally challenged with gliadin, revealed that Ivacaftor is highly effective in preventing gliadin-induced IL-15 upregulation, inflammasome activation, increase of intestinal permeability. Notably, Ivacaftor prevents the upregulation of IL-17, IL-21, IFN-γ induced by gliadin exposure in the small intestine of gliadin-sensitive mice and, instead, increases the production of TGF-β and IL-10 [27].

These pre-clinical results suggest that CFTR potentiators might be a possible therapeutic strategy in CD. However, translating preclinical data into clinical application requires prior validation in an appropriate disease context. To address this issue, peripheral blood mononuclear cells (PBMC) freshly collected from celiac individuals were co-cultured with intestinal epithelial cells to mimic a mucosal environment. Importantly, Ivacaftor prevented the production of IFN-γ induced by gliadin exposure while increasing the production of IL-10. Notably, such a preventive effect of Ivacaftor against gliadin-induced immunopathology could be correlated with its ability to potentiate CFTR function [27].

Altogether these findings demonstrate that gliadin-induced CFTR malfunction is at the apex of the pathogenic cascade leading to CD [27, 30]. Indeed, Ivacaftor-mediated maintenance of CFTR function was able to avoid the all gliadin induced manifestations of CD, both in mouse models of gluten enterophagy and in cells freshly collected from celiac individuals [27].

## Conclusion

Although at present the gluten-free diet is the sole option to avoid gluten related morbidity and prevent gluten-associated pathologies, other strategies are emerging. These still experimental approaches focus on enzymes that can degrade pathogenic gliadin peptides, thus reducing gliadin “toxicity”, attempt to prevent the intestinal permeabilization induced by gliadin, or aim at desensitizing celiac patients by means of “tolerogenic” vaccines [31–35]. In our recent work, we demonstrate a primordial pathogenic role for CFTR inhibition in CD. This discovery may open novel perspectives for the cure of celiac patients, suggesting that pharmacological stimulation of CFTR may interfere with a decisive step in the pathogenesis of CD. It remains to be evaluated whether CFTR potentiators may be used for the treatment of CD or whether they should rather be used for CD prevention. Moreover, it will be important to develop CFTR-potentiating agents that are less expensive than the drugs used for the treatment of CF. Thus, further efforts are required to identify natural compounds, repurposed drugs or new chemical entities endowed with the ability of potentiate CFTR channel gating.

## Abbreviations

BECN1: Beclin 1
CD: Celiac disease
CF: Cystic Fibrosis
CFTR: Cystic fibrosis transmembrane conductance regulator
FDA: Food and Drug Administration
HLA: Human leukocyte antigen
IFN-γ: Interferon gamma
IL-15: Interleukin-15
IL-17A: Interleukin 17A
IL-21: Interleukin 21
NBD1: Nuclear binding domain 1
NF-κB: Nuclear factor kappa B
NOD mice: Non-obese diabetic mice
ROS: Reactive oxygen species
TGM2: Tissue transglutaminase 2
VX-770: Ivacaftor
